# Transgene Pyramiding of Salt Responsive Protein 3-1 (*SaSRP3-1*) and *SaVHAc1* From *Spartina alterniflora* L. Enhances Salt Tolerance in Rice

**DOI:** 10.3389/fpls.2018.01304

**Published:** 2018-09-12

**Authors:** Hanamareddy Biradar, Ratna Karan, Prasanta K. Subudhi

**Affiliations:** ^1^School of Plant, Environmental, and Soil Sciences, Louisiana State University Agricultural Center, Baton Rouge, LA, United States; ^2^Department of Agronomy, University of Florida, Gainesville, FL, United States

**Keywords:** abiotic stress, halophyte, *Oryza sativa*, salt responsive protein 3-1 gene, smooth cordgrass, transgene stacking, vacuolar H^+^-ATPase subunit c1 gene

## Abstract

The transgenic technology using a single gene has been widely used for crop improvement. But the transgenic pyramiding of multiple genes, a promising alternative especially for enhancing complexly inherited abiotic stress tolerance, has received little attention. Here, we developed and evaluated transgenic rice lines with a single Salt Responsive Protein 3-1 (*SaSRP3-1*) gene as well as pyramids with two-genes *SaSRP3-1* and Vacuolar H^+^-ATPase subunit c1 (*SaVHAc1*) derived from a halophyte grass *Spartina alterniflora* L. for salt tolerance at seedling, vegetative, and reproductive stages. The overexpression of this novel gene *SaSRP3-1* resulted in significantly better growth of *E. coli* with the recombinant plasmid under 600 mM NaCl stress condition compared with the control. During early seedling and vegetative stages, the single gene and pyramided transgenic rice plants showed enhanced tolerance to salt stress with minimal wilting and drying symptoms, improved shoot and root growth, and significantly higher chlorophyll content, relative water content, and K^+^/Na^+^ ratio than the control plants. The salt stress screening during reproductive stage revealed that the transgenic plants with single gene and pyramids had better grain filling, whereas the pyramided plants showed significantly higher grain yield and higher grain weight compared to control plants. Our study demonstrated transgenic pyramiding as a viable approach to achieve higher level of salt tolerance in crop plants.

## Introduction

The productivity of rice (*Oryza sativa* L.) and other food crops is severely affected by abiotic stresses such as drought, salinity, flooding, temperature extremes, and nutrient deficiency or toxicity worldwide. Among these, soil salinity is a major abiotic stress negatively affecting the growth and productivity of rice and other major agricultural crops. Improving salt tolerance of crop plants and thereby minimizing yield loss is of paramount importance for any crop improvement program.

During last few decades, considerable efforts have been made through classical breeding to introgress genes from salt tolerant donor parents as well as wild relatives. As a result, commercial rice varieties have been improved through introgression of salt tolerance genes from promising landraces like Nona Bokra and Pokkali (Gregorio et al., 2002). However, major obstacles faced by plant breeders are the transfer of many undesirable attributes linked to the targeted salt tolerance genes (Thomson et al., 2010) and narrow genetic variation for salt tolerance in rice germplasm (Pearson and Bernstein, 1959; Kaddah, 1963; Lutts et al., 1995). Likewise, quantitative trait loci (QTL) mapping and modern genomics tools have been exploited for a better understanding of salt tolerance mechanisms as well as the development of new varieties. Several QTLs associated with salt tolerance traits have been identified in rice using several mapping populations derived from salt tolerant genotypes (Zhang et al., 1995; Koyama et al., 2001; Lin et al., 2003; Thomson et al., 2010). Similarly, dissection of salt tolerance through genome-wide association study (GWAS) revealed candidate salt tolerance-related genes in rice (Yu et al., 2017) and provided the platform for their functional analysis of salt tolerance. Since salt tolerant traits are controlled by many genes and influenced by environmental interactions (Flowers et al., 2000; Koyama et al., 2001; Lin et al., 2003), the development of salt tolerant varieties through marker assisted selection (MAS) requires extensive genotyping efforts and longer time to introgress many desirable QTLs into elite cultivars. Therefore, transgenic approach using novel salt tolerant genes from halophytes offers a promising alternative to augment the limited natural variation for salt tolerance attributes available in the rice germplasm. Smooth cordgrass is a halophytic grass species widely grown in coastal salt marsh areas of the United States. It can grow well in areas, where salinity is a major obstacle for plant growth. It can tolerate salt concentration up to 0.6 M NaCl (Vasquez et al., 2006). Since halophytes have evolved physiological mechanisms to survive under natural saline conditions, these species could be considered as a potential reservoir for isolating salt tolerance genes. During the last decade, extensive efforts have been made to isolate and characterize salt tolerant genes from halophytes like smooth cordgrass (Baisakh et al., 2008), *Thellungiella salsuginea*, a close relative of Arabidopsis (Wu et al., 2012; Ellouzi et al., 2014), *Salicornia europaea* L. (Lv et al., 2017), and *Brachypodium distachyon* (Takahashi et al., 2017). Recently, transgenic rice plants overexpressing a soluble inorganic pyrophosphatase gene *ThPP1* of *Thellungiella halophila* showed enhanced tolerance to alkaline stress compared to the wild type (He et al., 2017).

Generally, the plasma membrane is a primary structure which helps to maintain the intracellular ion homeostasis (Lv et al., 2017), and it selectively allows the exchange of other organic compounds. Salt stress is known to alter the structure and composition of membrane proteome, and thereby affecting the stability and solubility of essential components of membrane proteins (Katz et al., 2007). The PMP3 protein from *Saccharomyces cerevisiae* was first reported to be involved in salt tolerance (Navarre and Goffeau, 2000) followed by the discovery of its homolog *RCI2A* in Arabidopsis (Mitsuya et al., 2005) and *AcPMP3-1* in sheep grass (*Aneurolepidium chinense*). These genes helped to improve salt tolerance by restricting excessive uptake of Na^+^ (Inada et al., 2005).

Previous studies from the analysis of expressed sequence tags (ESTs) from salt stressed tissues demonstrated that *Spartina alterniflora* L. is a rich repository of salt stress-related genes (Baisakh et al., 2008) and indicated enhanced expression of plasma membrane protein3 (*SaPMP3*) and other unknown plasma membrane proteins under salt stress. In the present study, we evaluated the role of a novel salt responsive protein, *SaSRP3-1* in enhancing salt tolerance in rice through transgenic approach. The SaSRP3-1 protein has a single transmembrane domain which is a typical characteristic of plasma membrane proteins (Singh and Mittal, 2016). In addition to single gene transgenic lines, pyramided transgenic rice lines with both *SaSRP3-1* and vacuolar ATPase Subunit c1 gene (*SaVHAc1)* were generated and evaluated for salt tolerance at various stages of crop growth.

## Materials and Methods

### Plant Materials

Cocodrie, a high yielding long grain rice variety released by the Louisiana State University Agricultural Experiment Station (Linscombe et al., 2000), was used for *Agrobacterium* transformation.

### Selection of *SaSRP3-1* and *SaVHAc1* Genes

An EST sequence from *S. alterniflora* L. (GenBank Acc. No. EH277327) corresponding to Salt Responsive Protein3-1 (Sa*SRP3-1*) gene, highly expressed under salt stress condition (Baisakh et al., 2008), was used to develop transgenic rice plants. The complete open reading frame (ORF) of Sa*SRP3-1* from the *S. alterniflora* root cDNA library was amplified with primers SRP3-1F and SRP3-1R (**Supplementary Table S1**) for cloning into plant expression vector pCAMBIA 1305.2. In addition to single gene transgenic lines, pyramided transgenic rice lines were generated by crossing the homozygous *SaSRP3-1* plants with the previously developed transgenic plants overexpressing *SaVHAc1* from *S. alterniflora* L. (Baisakh et al., 2012).

### Overexpression of *SaSRP3-1* in *Escherichia coli* and Liquid Growth Assay

**FIGURE 1:**
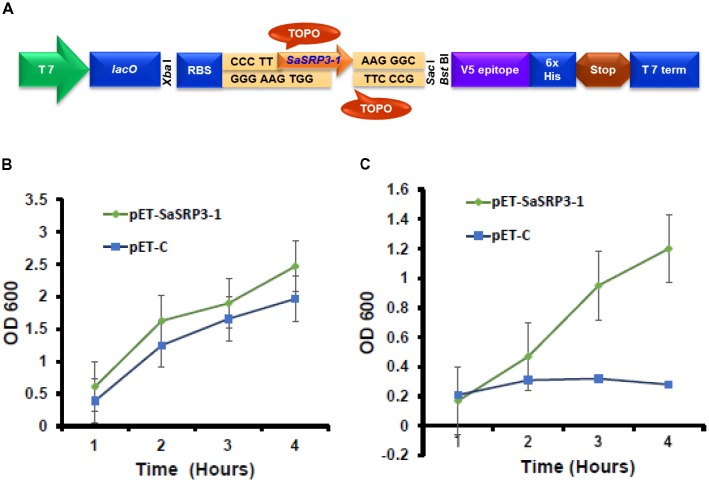
Cloning of *SaSRP3-1* into *E. coli* expression vector pET101/D-TOPO^®^. **(A)** Schematic representation of pET101/D-TOPO vector with cloning site, **(B)** Differences in the growth response curve of *E. coli* cells grown in the absence of NaCl in liquid LB culture. **(C)** Growth of *E. coli* cells after adding 600 mM NaCl to liquid LB media SaSRP3-1: pET-SaSRP3-1, ExCt1: pET-C. The mean of three independent measurements (OD_600_) are indicated at different time intervals.

For *Escherichia coli* overexpression, blunt-end PCR products from the complete ORF of *SaSRP3-1* was directionally cloned by adding four bases to the forward primer (CACC) into pET101/D-TOPO^®^ vector (Life Technologies, Grand Island, NY, United States). The gene was cloned after T7 promoter, which was induced in response to isopropyl β-D-1-thiogalactopyranoside (IPTG). The resulting recombinant vector was referred to as pET-SaSRP3-1 (**Figure 1A**). All primers used in this study for cloning, reverse transcription PCR, and confirmation of transgenic plants are listed in **Supplementary Table S1**. The recombinant plasmid pET-SaSRP3-1 as well as the plasmid pET-C, without the target gene, was transformed into competent *E. coli* strain BL21 (DE3). Transformed cells were inoculated in 3 mL of LB medium containing kanamycin (50 mg/L) and allowed to grow overnight in a shaker. The secondary culture was then initiated in 20 mL LB medium containing kanamycin (50 mg/L) with an equal concentration of each transformed cells at 37°C with constant shaking (250 rpm). In order to induce the protein synthesis in BL21 (DE3) cells, IPTG (100 mM) was added to the media when the OD_600_ value reached 0.5. For salt tolerance screening, BL21 (DE3) cells transformed with *SaSRP3-1* and expression control were streaked on selection media plates with varying salt concentration of 200, 400, 600, 800, and 1000 mM NaCl and incubated at 37°C. Based on the relative growth pattern, threshold NaCl concentration was determined to differentiate the growth of expression between control and recombinant cells.

For liquid growth assay, a secondary culture was initiated in 50 mL LB medium following the same procedure as described above and after induction of protein expression, 600 mM NaCl was added to LB broth in one flask but not to the control flask. The experiment was conducted in three replications, and the optical density of the culture was noted after a regular time interval (1, 4, 8, and 16 h) to construct the growth curve.

### Development of Transgenic Rice Plants

**FIGURE 2:**
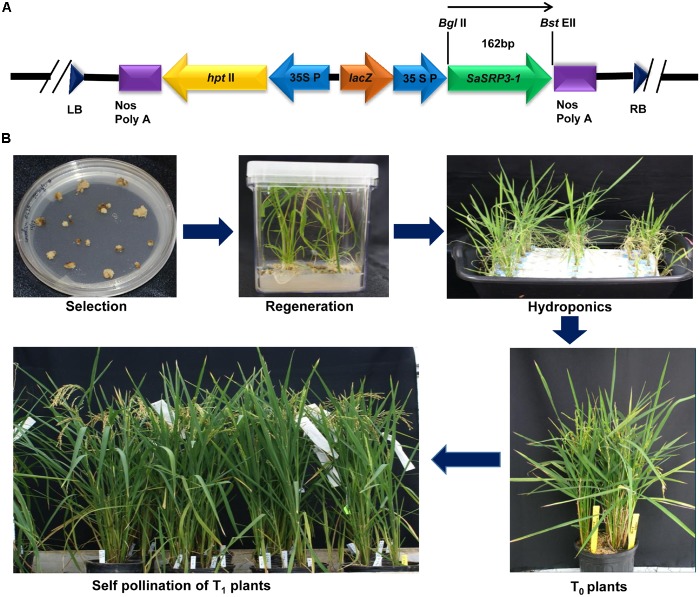
Plasmid construction for the gene *SaSRP3-1* and development of transgenic plants. **(A)** The schematic diagram of the partial linear plasmid vector and position of the gene *SaSRP3-1*. The diagram depicts the details of the pCAMBIA1305.2 vector containing right and left borders, *SaSRP3-1* gene between *Bgl*II and *Bst*EII sites, 35S CaMV promoter, plant selection marker (*hptII*), multiple cloning sites, and termination sites; **(B)** Transformation and regeneration of Cocodrie-*SaSRP3-1* transgenic rice plants showing selection, regeneration, and self-pollination of transgenic plants after *Agrobacterium* mediated transformation.

The pCAMBIA 1305.2 (CAMBIA, Australia), a binary plasmid vector showing stable replication in *E. coli* and *Agrobacterium tumefaciens*, was used to deliver the *SaSRP3-1* gene into embryonic calli. The plasmid contains the hygromycin B phosphotransferase (*hptII*) antibiotic resistance gene to screen the positive plants and kanamycin (*nptIII*) resistance gene for *E. coli* and *A. tumefaciens* selection (Broothaerts et al., 2005). The *A. tumefaciens* strain harboring pTOK233 plasmid and the virulence loci necessary for T-DNA transfer was used for callus infection (Hiei et al., 1994, 1997). Full-length cDNA clone of *SaSRP3-1* was cloned under the control of CaMV *35* promoter in pCAMBIA 1305.2 vector at *Bgl*II and *Pml*I restriction endonucleases sites (**Figure 2A**). The plant expression vector pC-SaSRP3-1 was confirmed by both restriction analysis and DNA sequencing and then transformed into *A. tumefaciens* strain LBA4404 (Hoekema et al., 1983) for rice calli infection.

Dehusked rice seeds from Cocodrie variety were used for callus induction following the method of Hiei et al. (1994). Four to six weeks old light yellowish and compact embryogenic calli were used for *Agrobacterium* transformation. After *Agrobacterium* co-cultivation, calli were placed on selection media with hygromycin (50 mg/L) and 3–4 cycles of selection were performed. Subsequently, these calli were transferred to regeneration media and then into the rooting medium (**Figure 2B** and **Supplementary Table S5**). After 5 days of root regeneration, small seedlings of 3 to 8 cm height were initially grown in hydroponics under control conditions of light with six 40W Sylvania cool white fluorescent bulbs (∼100 μE m^−2^ s^−1^) at 26°C in 1/4th strength Yoshida media (Yoshida et al., 1976). The hydroponic nutrient media was replaced with fresh media after every 7 days. Transgenic plants were identified by PCR amplification of the target gene as well as hygromycin B phosphotransferase gene. The positive seedlings were grown in the greenhouse and self-pollinated to advance the generation (**Figure 2B**). The greenhouse growing condition was set at 13:11 h (day: night) photoperiod with the temperature setting of at 28°C/25°C (day/night) and a light intensity of 350 μE m^−2^ s^−1^. Five seedlings were allowed to grow per 1-gallon pot of 6.25-inch depth × 6.5-inch diameter size.

### Molecular Confirmation of Transgenic Plants

Regenerated individual plants from hydroponic media at 4–5 leaf stage were analyzed to confirm the successful transformation. Transgenic plants were identified by PCR amplification of *SaSRP3-1* and *hptII* genes by gene specific primers in T_0_ generation. The positive transgenic plants were then transferred to greenhouse and selfed for two successive generations to get homozygous plants. All PCR amplification reactions were performed using the protocol of Baisakh et al. (2008).

Transgenic rice plants selected by PCR analysis were further confirmed through Southern hybridization (Sambrook and Russell, 2001). Ten micrograms of genomic DNA from independent positive plants as well as untransformed control plants was digested with *Hind*III (Fermentas, Waltham, MA, United States), which cuts at a single restriction site within the T-DNA region (pCAMBIA 1305.2). The separated DNA on agarose gel was transferred to Hybond^TM^ N^+^ membrane (GE Healthcare Biosciences, Piscataway, NJ, United States) following standard procedure and DNA on blot was fixed by UV cross-linking.

Southern hybridization was done using the ECL direct nucleic acid labeling and detection system (GE Healthcare Biosciences, Piscataway, NJ, United States). Transformed *SaSRP3-1* gene present on T-DNA was used as a probe to detect the transgenic plants The blots were exposed to autoradiography film (Hyperfilm ECL, GE Healthcare, Piscataway, NJ, United States) for 5 h to visualize the gene specific bands (**Supplementary Figure S3**).

### Reverse Transcription PCR (RT-PCR)

Total RNA was isolated from T_2_ homozygous rice seedlings using the RNeasy Plant Mini Kit (QIAGEN, Valencia, CA, United States) and was eluted in RNase-free water for gel analysis and RT-PCR. First strand complementary DNA (cDNA) was synthesized using the qScript^TM^ Flex cDNA Synthesis Kit (Quanta BioSciences, Gaithersburg, MD, United States). The cDNA was subsequently used as a template for RT-PCR with gene specific primers. The amplification of elongation factor 1-α gene (*ef1α*) was used as an internal control for comparing gene expression in wild type and transgenic rice plants. The amplified products were separated on 1.2% agarose gel for comparison of gene expression.

### Phenotypic Characterization of Transgenic Plants

For phenotypic characterization T_2_ homozygous *SaSRP3-1*, T_3_ homozygous *SaVHAc1*, and homozygous pyramided plants with two-genes *SaSRP3-1* and *SaVHAc1* were used for salt stress screening at seedling, vegetative, and reproductive stages. For seedling screening, rice seeds were germinated in Petri plates and 5-day-old seedlings were transferred to Yoshida nutrient media (Yoshida et al., 1976) under a 14 h light and 10 h dark at 25°C. The seedlings were supported on a styrofoam sheet fabricated with supporting sponge or nylon mesh at the bottom. Seedlings were allowed to grow under normal condition for 10 days. Salt stress was imposed to 12-day-old seedlings by adding 100 and 150 mM NaCl to the nutrient solution and pH 5.2 was maintained. The control plants were not subjected to salt stress. Plant growth related traits such as shoot and root length were measured on five seedlings both before and 12 days after stress treatment for comparison. For vegetative stage screening, plants were exposed to 60 and 80 mM NaCl 45 days after planting. Physiological observations (chlorophyll content, relative water content [RWC], and proline content) were recorded 12 days after salt stress under greenhouse condition. The unstressed leaves at the vegetative stage were used for leaf disk assay.

The wild type and transgenic plants were used for salinity tolerance screening at reproductive stage under greenhouse conditions by adding 60 mM NaCl at the time of boot leaf emergence. During both vegetative and reproductive stage screening, fresh salt solution was added regularly at 3 days intervals to maintain the desired salt stress and pH 5.2. For reproductive stage screening, salt stress and pH were maintained until harvesting. Phenotypic traits such as grain yield per plant, 100-grain weight, and seed sterility (%) were recorded. The 100-grain weight was measured on 100 seeds after drying at 45°C in an oven for 5 days after harvesting. The number of filled and chaffy spikelets was counted at the time of harvesting and seed sterility percentage was computed as follows.

$$\mathrm{Seed sterility}\;(\%)=\frac{\mathrm{Number of unfilled spikelets}}{\mathrm{Total number of spikelets}}\times 100$$

### Leaf Disk Assay

Leaf injury due to leakage of ion and damage to the photosynthetic unit was visually evaluated by floating leaf disk assay. During vegetative stage, fully expanded leaf sections of 2 cm length from one leaf below the flag leaf were used for comparison. Leaf disks were floated in control and NaCl concentrations of 100 mM and 150 mM. After 72 h, the phenotypic difference in chlorophyll retention was visually compared (Munns and James, 2003; Baisakh et al., 2012).

### Measurement of Chlorophyll Content

The chlorophyll content was measured to see the possible differences between transgenic plants and control plants. Leaf samples from vegetative stage screening were used for comparing the variation in total chlorophyll content. Fresh leaf weight was measured, and chlorophyll was extracted with 80% acetone from individual transgenic and control plants. The absorbance of the supernatant was measured at 645 and 663 nm. The concentration of chlorophyll a (Chl-a), chlorophyll b (Chl-b), and total chlorophyll (Chl-t) were estimated using the extinction coefficient equations (Arnon, 1949). Total chlorophyll content in leaf sample was expressed as milligram per gram of fresh leaf weight (mg/g).

$$\begin{array}{l} \text{chl}-\text{a}=12.72{\text{ A}}_{663}-2.59{\text{ A}}_{645}\\ \text{chl}-\text{b}=22.9{\text{ A}}_{645}-4.67{\text{ A}}_{663}\\ \text{chl}-\text{t}=20.31{\text{ A}}_{645}+8.05{\text{ A}}_{663} \end{array}$$

### Relative Water Content (RWC)

A leaf section of 2 cm length from one leaf below the flag leaf was used to determine the RWC during vegetative stage. Ten leaf sections of 2 cm length were collected 5 cm below the leaf tip from each plant and fresh weights were taken. The leaf sections were then transferred to fresh Yoshida nutrient solution (Yoshida et al., 1976) for about 3 h at room temperature under dark condition and turgid weight was taken. Leaf samples were dried in oven at 70°C overnight and dry weights were taken. The RWC was calculated using the following formula.

$$\text{RWC}(\%)=\frac{\left(\text{FW}-\text{DW}\right)}{\left(\text{TW}-\text{DW}\right)}\times 100$$

where FW is fresh weight, DW is dry weight, and TW is turgid weight.

### Proline Estimation

Proline accumulation in response to hyperosmotic stress was well documented in Arabidopsis (Toka et al., 2010) and other plants. The amount of accumulated proline in transgenic and control plants was quantified during the vegetative stage after exposure to salt stress (60 and 80 mM NaCl) and control conditions following the procedure of Bates et al. (1973). Known quantity of leaf samples were homogenized in 3% 5-sulfosalicylic acid. The amount of proline content in each sample was quantified by ninhydrin labeling. Total amount of proline content in the leaf sample was expressed as microgram per gram of fresh leaf weight (μg/g).

### Measurement of Na^+^ and K^+^ Content

Total Na^+^ and K^+^ content in shoots were measured under stress and control conditions during seedling stage salt stress. Leaf samples were collected and rinsed thoroughly in distilled water to ensure the exclusion of external salt deposition. Samples were completely dried at 80°C for 2 days and powdered. One hundred micrograms of powdered samples were incubated with 5 mL of 4 M HCl at 37°C overnight to extract Na^+^ and K^+^ (Zhao et al., 2007). The supernatant was filtered, and diluted samples were used to determine the total Na^+^ and K^+^ by a Flame photometer (Jenway Model PFP7, Bibby Scientific Limited, United Kingdom). Total concentration of Na^+^ and K^+^ in plant samples were estimated based on the concentration determined in NaCl and KCl standard curve and, K^+^/Na^+^ ratio was used for comparing the transgenic and control plants.

### Statistical Analysis

The effect of salt stress on various morphological and physiological traits was analyzed by one-way ANOVA using the mean observations from replicated experiments separately for salt stress and control condition. Differences in mean values of traits for transgenic and control plants were analyzed using paired *t*-test. *Post hoc* comparison was made using Dunnett’s test, when one-way ANOVA results showed the difference in genotypic mean values at 0.05 probability level. All statistical analyses were performed using SAS version 9.3 for Windows (SAS Institute, 2011).

## Results

### Nucleotide and Amino Acid Sequence Analysis

The ORF of the EST *SaSRP3-1* (Baisakh et al., 2008) was 162 bp long and the protein was made up of 54 amino acids (**Supplementary Figures S1A,B**). The secondary structure prediction indicated the presence of one prominent α helix structure and very small size β strand as well (**Supplementary Figure S1C**). Amino acid analysis indicated that 44 amino acids were hydrophobic in SaSRP3-1 and 14 to 28th amino acids formed a single hydrophobic transmembrane domain (TMD) (**Supplementary Figure S1D**). Moreover, comparison of its nucleotide sequence to accessions in the non-redundant sequence database using BLASTN interface indicated 94–95 percent similarity to stress induced hydrophobic protein PMP from other plant species (**Supplementary Table S2**). The presence of a single TMD indicated the possibility of unique PMP evolution in *S. alterniflora* to form an uncharacterized but crucial protein to survive under abiotic stress environments.

### Overexpression of *SaSRP3-1* in *E. coli*

The results from the *SaSRP3-1* overexpression experiment in *E. coli* indicated normal bacterial colony growth under control, 200, and 400 mM NaCl concentrations and there was no significant difference in growth between the pET-SaSRP3-1 transformed and control *E. coli* cells (**Supplementary Figure S2**). Since control condition, 200 and 400 mM NaCl concentrations did not differentiate the growth of *SaSRP3-1* transformed *E. coli* from *E. coli* cells without transgene, we observed similar pattern of colony growth at these NaCl treatments. Interestingly, more colony growth was observed at 600 mM NaCl only with pET-SaSRP3-1 transformed *E. coli* cells compared to expression control *E. coli* cells (pET-C). The critical difference between the growth of transformed and control *E. coli* was found only at 600 mM NaCl, where the transformed *E. coli* cells showed a gradual increase in growth with maximum growth difference 4 h after salt stress. Therefore, above results implies that *SaSRP3-1* contributed toward salt tolerance in *E. coli* at 600 mM NaCl and at low salt stress, up to 400 mM NaCl, intrinsic mechanism of *E. coli* cells supported normal growth, which was not observed at 600 mM NaCl and higher level of salt stress. However, the growth of both transformed and control cells ceased at NaCl concentrations beyond 600 mM. This scenario implies that *SaSRP3-1* was contributing for salt tolerance at 600 mM, whereas higher salt concentration seems to be detrimental.

To validate how this above observation is translated into realistic growth pattern, the quantitative growth difference at 600 mM NaCl was measured using liquid growth curve assay, which is more accurate. The growth of bacterial cells in terms of optical density (OD 600 nm) was plotted at different time intervals to examine the relationship between protein expression and salt tolerance. Optical density (OD 600 nm) observations revealed that even under control condition, there were differences in the growth of *E. coli* cells transformed with expression vector control and cells transformed with *SaSRP3-1* (**Figure 1B**). Moreover, the optical density (OD) of the cells transformed with *SaSRP3-1* was more than the expression control and the magnitude of this effect increased with incubation time. This observation indicated that SaSRP3-1 protein has little influence on the growth of *E. coli* cells under control condition. But when these cells were grown under 600 mM NaCl and their OD_600_ were compared, there was a significant difference between the growth of *E. coli* cells transformed with *SaSRP3-1* and cells transformed with expression control vector at several time points (**Figure 1C**). Moreover, cells transformed with *SaSRP3-1* showed normal growth curve pattern at 600 mM NaCl, whereas *E. coli* cells transformed with expression control vector did not grow normally. We also observed faster growth of transformed *E. coli* cells immediately after approximately 1 h of salt exposure, which is the characteristic of a log phase of growth. Based on this *E. coli* expression results, it can be inferred that *SaSRP3-1* plays an important role in the adaptation of transformed *E. coli* cells under salt stress.

### Molecular Analysis of Transgenic Rice

**FIGURE 3:**
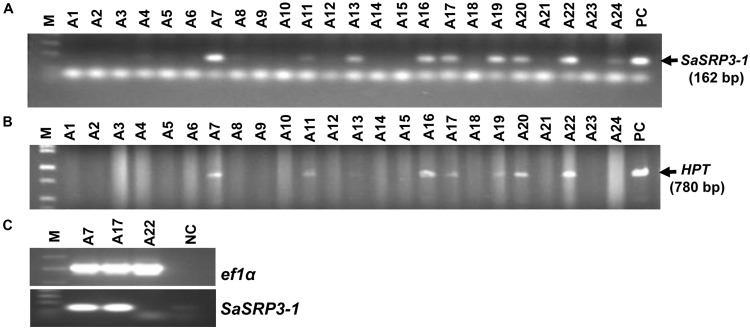
Confirmation of *SaSRP3-*1 containing T_0_ transgenic rice plants by PCR and detection of *SaSRP3-1* mRNA by semi-quantitative RT-PCR analysis of transgenic seedlings grown in greenhouse condition. **(A)** PCR confirmation using *SaSRP3-1* specific primers; **(B)** PCR confirmation of T_0_ plants using hygromycin phosphotransferase (*hptII*) specific primers. M: 1 Kb DNA ladder, A1 to A24: DNA isolated from individual T_0_ transgenic plants, PC: Positive control; **(C)** a representative RT-PCR experiment for detection of expression of elongation factor 1-α (*ef1α*) and *SaSRP3-1* mRNA in seedling stage leaf samples of transgenic and Cocodrie (WT) plants. M: 1 Kb ladder, A7, A17, and A22: transgenic plants with *SaSRP3-1* gene, NC: Negative control without any sample.

**FIGURE 4:**
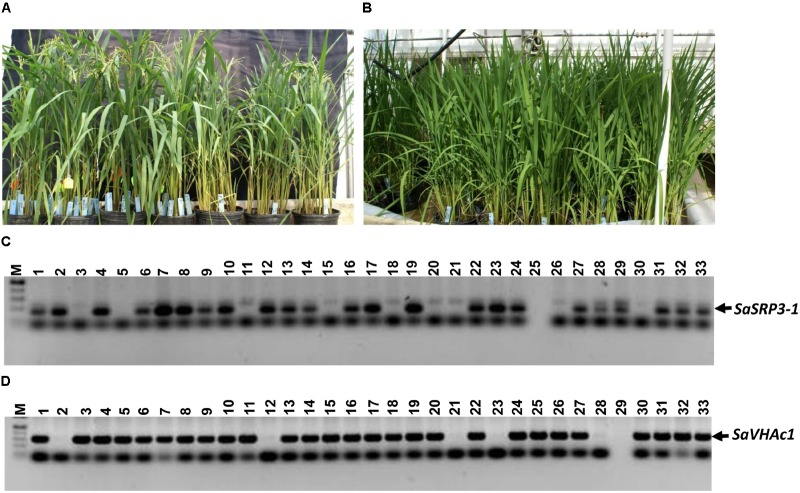
Transgenic plants grown in green house and PCR analysis to confirm the integration of *SaSRP3-1* and *SaVHAc1* genes in pyramided progenies. **(A)** Pyramiding of *SaSRP3-1* (T_1_) and *SaVHAc1* (T_2_) genes by crossing homozygous plants during flowering stage; **(B)** Growth of pyramided progenies with both genes; **(C)** PCR amplification showing the presence of *SaSRP3-1* gene; **(D)** Pyramided transgenic rice plants showing the amplification of *SaVHAc1* gene using gene specific primers.

Seven independent transgenic rice lines were obtained using the *Agrobacterium*-mediated transformation method, and the transformation efficiency was 29.2% (**Figures 3A,B**). Progenies from three independent transgenic plants were used for RT-PCR, and four independent transgenic plants were used for both Southern confirmation and phenotypic screening at different levels of salt stress. Transgenic rice lines generated after selection and regeneration were confirmed by PCR, RT-PCR, and Southern hybridization. As an initial step of molecular screening, T_0_ generation transgenic plants were confirmed by PCR amplification of *SaSRP3-1* and *hptII* genes by gene specific primers. PCR analysis confirmed the presence of *SaSRP3-1* and *hptII* genes in seven regenerated lines (**Figures 3A,B**). Subsequently, homozygous *SaSRP3-1* plants were obtained by selfing and then crossed with *SaVHAc1* transgenic plants to obtain pyramided plants carrying both genes (**Figures 4A,B**). The resulting two gene combination pyramided rice lines were confirmed by PCR amplification of both *SaSRP3-1* and *SaVHAc1* genes. Twenty-one plants were confirmed for the presence of both transgenes out of thirty-three progenies (**Figures 4C,D**). Further, southern hybridization results revealed the presence of multiple copies of *SaSRP3-1* in the selected transgenic lines (**Supplementary Figure S3**). The RT-PCR analysis showed expression of *SaSRP3-1* in the selected transgenic lines (**Figure 3C**). However, plant #A22 did not show amplification in RT-PCR despite being confirmed for the presence of *SaSRP3-1* based on regular PCR. It implies that plant #A22 did not actively express the *SaSRP3-1* transgenic gene. Post-transcriptional gene silencing influenced by various environmental factors could be the possible reason for non-detection of the transcript in RT-PCR.

### Salinity Tolerance Screening

#### Comparison of Shoot and Root Length

**FIGURE 5:**
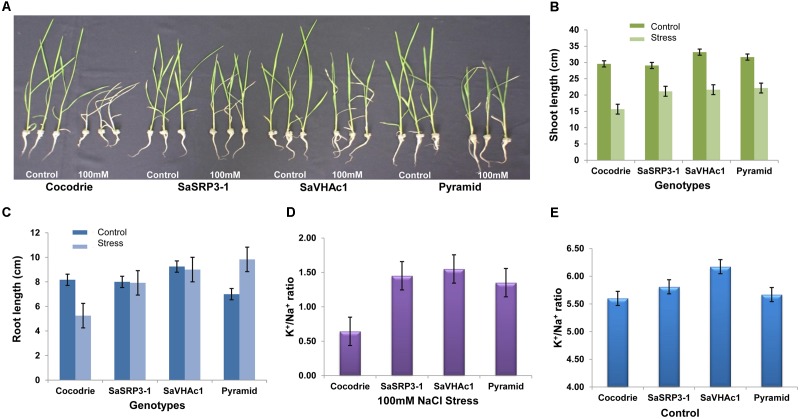
Salt tolerance evaluation of transgenic and wild type plants. **(A)** Responses of transgenic and wild type rice plants to 100 mM NaCl stress during seedling stage; **(B)** Comparison of shoot length; **(C)** Comparison of root length; **(D)** K^+^/Na^+^ ratio in shoot 12 days after exposure to 100 mM salt stress; **(E)** K^+^/Na^+^ ratio in shoot under control condition; Values represent means ± SD of five independent replicates. Error bars represent standard error.

Five-day old seedlings with uniform growth were transferred to hydroponic culture, and after 7 days of growth under the normal hydroponic condition, seedlings were transferred to different salt treatments (**Supplementary Figure S4**). Shoot and root lengths were compared after imposing different salt stress for 12 days. Notable changes in plant height were observed at 100 mM NaCl stress compared to the control condition (**Figure 5A**). All transgenic plants showed increased shoot growth compared to Cocodrie (WT) under 100 mM NaCl salt stress (**Figure 5B**). As expected, the growth of transgenic and WT seedlings was normal under control condition, and all plants had similar shoot length. But there was increase in the shoot length of transgenic plants compared to the WT under salt stress (**Supplementary Table S3**).

The root length of Cocodrie plants ceased to grow at 100 mM NaCl compared to their root growth under the control condition. In contrast, 100 mM NaCl did not negatively affect the root growth in transgenic plants carrying either *SaSRP3-1* or *SaVHAc1* gene. We observed the non-significant effect on root growth pattern between control and 100 mM NaCl stress condition in single gene transgenic plants. However, the growth of root was significantly higher in pyramided transgenic plants carrying both *SaSRP3-1* and *SaVHAc1* genes at 100 mM NaCl salt stress than the control condition (**Figure 5C**). These results showed that the root growth was not affected by salt stress in transgenic plants with either single or two transgenes, which accounts for the relative ineffectiveness of salt stress on root growth of transgenic plants compared to WT plants (**Supplementary Table S4**). This normal growth of roots observed in transgenic plants is a clear indication of salt tolerance level of *SaSRP3-1* and *SaVHAc1* genes. Moreover, the higher root growth found in pyramided plants could be a complementary effect of these two genes. However, analyses of seedling growth at 150 mM salt stress did not generate any valuable data as plants showed severe wilting symptoms immediately after 2 days of salt stress.

#### Estimation of Na^+^ and K^+^

Our results on Na^+^ and K^+^ content in shoot tissue indicated that *SaSRP3-1* overexpressing transgenic plants had significantly higher K^+^/Na^+^ ratio than non-transformed plants under salt stress condition (**Figure 5D**). Likewise, the transgenic plants with *SaVHAc1* and pyramided transgenic plants with both genes had significantly higher K^+^/Na^+^ ratio in shoot tissues than WT plants under 100 mM NaCl salt stress. The shoot tissue had more available K^+^ in all transgenic plants compared to control plants. In contrast, leaf K^+^ and Na^+^ ratio under control condition did not show any significant difference between single gene transgenic lines and WT. However, only *SaVHAc1* transgenic plants had significantly higher leaf K^+^/Na^+^ ratio than others under control condition (**Figure 5E**).

#### Leaf Disk Salt Assay and Chlorophyll Content

**FIGURE 6:**
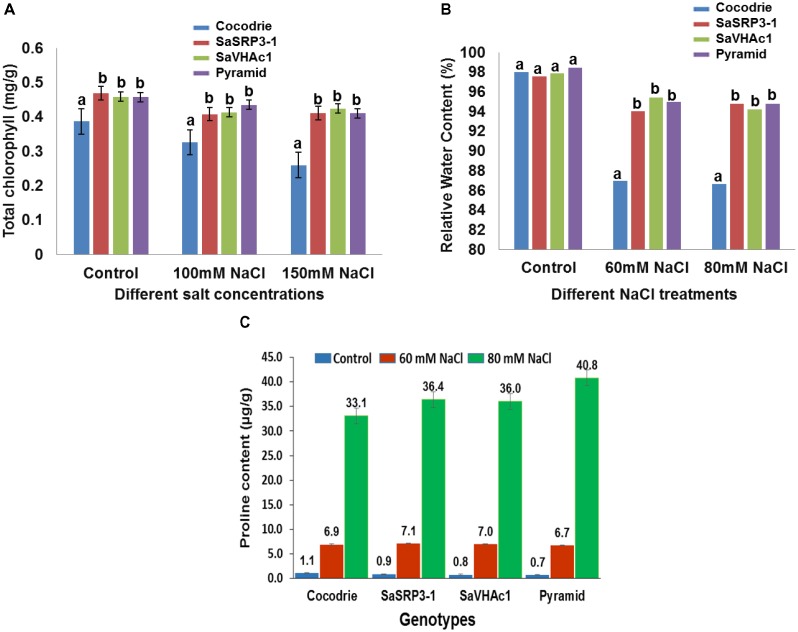
Physiological analysis of Cocodrie, single gene (*SaSRP3-1 and SaVHAc1)* and pyramided transgenic rice plants. **(A)** Total chlorophyll content in leaf samples from different transgenic and Cocodrie (WT) plants 4 days after salt stress (100 and 150 mM NaCl); **(B)** Comparison of relative water content (RWC) under control and salt stress (60 and 80 mM NaCl) conditions; **(C)** Proline accumulation in transgenic and WT plants under salt stress (60 and 80 mM NaCl) 10 days after salt stress. Error bars represent standard error. Different letters on bar diagram indicates statistical significance at 5% level based on Dunnett’s test.

The leaf disk assay is a sensitive and rapid technique for preliminary identification of salt tolerant plants. In the present investigation, it was used to assess the extent of chlorophyll retention under varying salt stress conditions. Based on visual observation, there was no visible difference between transgenic and WT plants under control condition (**Supplementary Figure S5**). However, transgenic plants over-expressing *SaSRP3-1, SaVHAc1*, and pyramided line with two genes retained more chlorophyll compared to Cocodrie (WT) at 100 mM and 150 mM NaCl salt stress (**Figure 6A**). This implies that loss of chlorophyll was less in transgenic plants under salt stress. Since this assay is still a qualitative approach, the actual quantity of total chlorophyll content in leaves was measured to confirm the normal functioning of photosynthetic activity at a physiological level. The total chlorophyll content of transgenic plants was least affected under salt stress compared with the control plants.

#### Relative Water Content (RWC)

Relative water content has proven to be a valuable index for measuring leaf turgidity, which is known to be affected by salt stress at initial stages of salt stress. There was clearly retention of more water in transgenic rice plants compared to Cocodrie (**Figure 6B**). There was no significant difference in RWC between transgenic and WT plants under control conditions. However, RWC was significantly higher in transgenic plants with single and two gene combinations at 60 and 80 mM NaCl compared to WT. These observations suggested that the transgenic plants maintained normal leaf turgidity and photosynthetic function under salt stress condition. Although we evaluated the plants at 60, 80, 100, and 150 mM NaCl conditions, critical difference was found only at 60 and 80 mM NaCl concentrations. As the higher salt stress of 100 and 150 mM NaCl treatments showed the severe loss in RWC in both WT and transgenic plants, the results were not presented.

#### Free Proline Content

Accumulation of proline, an organic compatible osmolyte, in response to salt stress was measured 10 days after exposure to NaCl stress using ninhydrin method. All plants showed an average of 7-fold increase in proline accumulation at 60 mM NaCl compared to the control condition, and there was no difference between Cocodrie and transgenic plants. Further, when salt stress was increased to 80 mM NaCl, there was 33-fold, 36-fold, and a 40-fold increase in free proline accumulation in leaf compared to the control condition in Cocodrie, single gene transgenic plants, and pyramided plants, respectively (**Figure 6C**). Transgenic plants accumulated more proline content at 80 mM NaCl stress than control plants. The single gene and pyramided transgenic plants accrued 9 and 21% more proline at 80mM salt stress compared to the control condition, respectively. But the increased accumulation was more in pyramided plants at 80 mM NaCl stress compared to their single gene transgenic and WT plants. The significant increase of proline accumulation in pyramided plants was most likely conferred by the presence of two salt tolerance genes.

#### Yield Parameters in Transgenic Plants

**FIGURE 7:**
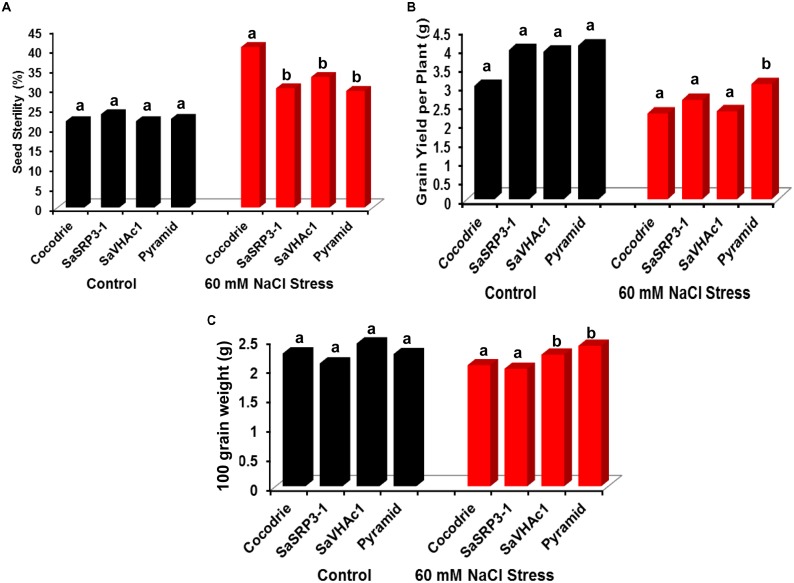
Analysis of yield parameters in transgenic and control plants under salt stress at the reproductive stage in greenhouse. **(A)** Impact of salt stress on grain filling and seed sterility; **(B)** comparison of grain yield per plant under salt stress; **(C)** variation in 100-grain weight due to salt stress. Different letters on bar diagram indicate the statistical significance at 5% level based on Dunnett’s test.

The transgenic plants were evaluated at the reproductive stage to determine the effect of salt stress on traits influencing yield. The percent seed sterility was calculated to explore the effect of salt stress on grain filling. As expected, there was no significant difference in seed sterility among the genotypes under the control condition (**Figure 7A**). In contrast, the seed sterility was significantly lower in all the plants carrying single transgene as well as pyramided plants with two gene combination than Cocodrie (WT) plants under 60 mM salt stress condition. The results indicated that plants carrying single transgene as well as pyramided plants could enhance salt tolerance at the reproductive stage.

There was no significant difference in grain yield per plant between transgenic and WT plants under control conditions (**Figure 7B**). But the grain yield was affected in WT as well as transgenic plants with single genes under 60 mM NaCl stress. However, only pyramided transgenic plants showed significantly higher grain yield per plant than others. Further, we found no significant difference in 100-grain weight between transgenic lines and Cocodrie under control condition (**Figure 7C**). However, upon imposing 60 mM salt stress, rice plants transformed with *SaVHAc1* and pyramided plants exhibited significantly higher 100-grain weight than WT and transgenic plants with *SaSRP3-1*.

## Discussion

Salt tolerance of plants is a complex trait due to the involvement of multiple genes associated with numerous mechanisms. The physiological responses to salinity depend on the phenology of the plants (Munns, 2002) and environmental influence (Flowers et al., 2000; Koyama et al., 2001; Lin et al., 2003). Although considerable efforts have been made to improve salt tolerance of field crops through conventional breeding as well as QTL approach using salt tolerant donors and wild relatives (Gregorio et al., 2002; Thomson et al., 2010), the progress in improving growth and yield parameters were not satisfactory under salt stress conditions. In this study, we demonstrated that overexpression of a halophytic gene *SaSRP3-1* as well as its combination with *SaVHAc1* from *S. alterniflora* L., in pyramided transgenic plants, could improve the salt tolerance at seedling, vegetative, and reproductive stages.

The amino acid sequence of SaSRP3-1 showed no homology to any of the functionally characterized protein in the NCBI non-redundant protein database. It has a hydrophobic transmembrane helix from 14th to 28th amino acid sequences. The presence of uncharacterized single transmembrane domain having a high homology of 94–95 percent to uncharacterized hydrophobic proteins (**Supplementary Table S2**) indicated that SaSRP3-1 is a novel salt-responsive protein. In addition, the presence of a transmembrane domain (TMD) based on the secondary protein structure suggests that SaSRP3-1 may be a subunit of big plasma membrane protein 3 (PMP 3) protein due to similarity of its TMD to the TMD2 of the PMP3. However, the exact function of the protein is not known. It is possible that *SaSRP3-1* may be the product of alternative splicing regulated either by histone modifications or non-coding RNAs. Recent advances in the next generation sequencing technologies have revealed the prevalence of alternative splicing across eukaryotic genomes leading to the production of multiple distinct functional transcripts from a single gene (Luco and Misteli, 2011; Walters et al., 2013).

Functional characterization of genes in microbial model organisms such as *E. coli* and yeast has been widely used for screening salt-sensitive mutations (Yamada et al., 2003). The liquid growth curve assay of *E. coli* cells transformed with *SaSRP3-1* showed that *E. coli* cells transformed with *SaSRP3-1* grew normally at 600 mM NaCl concentration, while expression control cells ceased to grow (**Figure 1C**). Therefore, *SaSRP3-1* may be involved in some cellular activity, which is crucial for survival under salt stress. This is the first line of evidence indicating the involvement of *SaSRP3-1* gene in salt tolerance mechanism. Previous studies proved the suitability of a similar *E. coli* overexpression study to test salt tolerance of mangrove allene oxide cyclase (*AOC*) and characterized the salt tolerance role of *AOC* in *E. coli*, yeast, and tobacco cells (Yamada et al., 2002). Apart from overexpression, another advantage of *E. coli* system is the feasibility of protein purification and subsequent enzymatic characterization. Liu et al. (2011) overexpressed ascorbate peroxidase (*APX*) gene from *Brassica napus* in *E. coli* BL21 (DE3) and demonstrated that enzymatic assay conducted from purified protein increased the activity of APX decompose H_2_O_2_ produced during salt stress.

Since some physiological traits are adversely affected under salt stress, it is necessary to understand how overexpression of *SaSRP3-1* influences vegetative growth and grain yield in rice. Transgenic lines selected in this study had multiple copies of *SaSRP3-1*. Interestingly, one out of three transgenic rice lines failed to show *SaSRP3-1* expression, which might be due to *SaSRP3-1* transgene silencing or epigenetic modification (Rajeevkumar et al., 2015). It was clearly demonstrated that the *SaSRP3-1* and pyramided transgenic plants retained significantly higher chlorophyll content and RWC under salt stress (**Figures 6A,B**). Additional evidence for enhancing salt tolerance in transgenic rice was substantiated by the maintenance of higher K^+^/Na^+^ during salt stress at the vegetative stage (**Figure 5D**). In contrast, control plants showed low RWC, increased bleaching of chlorophyll as well as the low K^+^/Na^+^ in leaves. Based on the research findings in yeast (Navarre and Goffeau, 2000), tomato (Žižková et al., 2015), Arabidopsis (Mitsuya et al., 2005; Karan and Subudhi, 2012a), and rice (Song et al., 2011), the decrease in total chlorophyll content and low RWC in Cocodrie can be attributed to toxicity caused by accumulation of more Na^+^ in WT plants. Consequently, these results infer that higher chlorophyll and higher RWC observed in transgenic plants could be due to the prevention of excess Na^+^ accumulation in root and shoot, which in turn helps to maintain ion homeostasis and normal cellular functions.

Excess accumulation of Na^+^ was toxic to cellular activities, and it may directly affect the photosynthetic activity through the reduction in chlorophyll content at cellular level. The bleaching of chlorophyll under salt stress has been reported by many researchers (Shrivastava et al., 2010; Karan and Subudhi, 2012b; Žižková et al., 2015; Srivastava et al., 2016). In addition, improvement in seedling growth and high K^+^/Na^+^ has been demonstrated in rice plants overexpressing *SaVHAc1* (Baisakh et al., 2012), *OsNAC5* (Song et al., 2011) and Arabidopsis plants overexpressing of *SaSce9* (Karan and Subudhi, 2012a). Likewise, the significant reduction of RWC in control plants suggests osmotic stress caused by salt stress, whereas transgenic plants showed tolerance to water loss during salt stress. The tolerance to water loss could be due to reduced stomatal openings in transgenic plants. Evidences for such higher RWC under salt stress has been reported in Arabidopsis plants overexpressing *AVP1*-*1* and *AVP1*-*2* (Gaxiola et al., 2001) and *SaARF1* (Karan and Subudhi, 2014) and rice plants overexpressing *SaVHAc1* (Baisakh et al., 2012) and transcription factor HYR (Ambavaram et al., 2014).

Proline accumulation has been one of the strategy to maintain the normal functioning of cells under different environmental stresses, such as drought, high salinity, and low temperature in plants (Sanchez et al., 2008; Usadel et al., 2008; Lugan et al., 2010; Karan and Subudhi, 2012b; Jaarsma et al., 2013). The results indicated a marked difference in the amount of proline accumulation between the transgenic and WT plants at 80 mM salt stress than the control condition. The magnitude of difference was more and distinct at 80 mM stress between transgenic and control plants than at 60 mM NaCl stress (**Figure 6C**) suggesting the role of proline as osmoprotectant during salt stress as well as in maintaining normal cellular functions (Karan and Subudhi, 2012b; Jaarsma et al., 2013). Furthermore, the higher proline accumulation by pyramided plants indicates that *SaSRP3-1* and *SaVHAc1* genes have a different mechanism of salt tolerance and together they are acting in a synergistic manner to increase the level of salt tolerance in pyramided plants. Alternatively, this result also implies the possibility of a decrease in protein synthesis and protein degradation during vegetative stage salt stress. The general increase in proline accumulation at 60 mM NaCl stress by all plants, including Cocodrie along with transgenic plants, supports the hypothesis that proline accumulation is an indication of the onset of salt stress as well as plants’ tolerance level.

Furthermore, both single and two-gene transgenic plants displayed significantly more shoot and root growth in response to salt stress during the early seedling stage. These transgenic plants not only survived from wilting and drying symptoms but also showed significantly enhanced shoot and root growth under salt stress (**Figures 5B,C**). The enhanced salt tolerance of pyramided lines could be attributed to V-ATPase, which is known to participate in the vacuolar Na^+^ compartmentation by regulating both vacuolar H^+^-ATPase (*V-ATPase*) and vacuolar H^+^-PPase (*V-PPase*) activity as demonstrated in the euhalophyte *S. europaea* (Lv et al., 2017). In contrast, we noticed a significant reduction in both shoot and root growth in wild type. The reduction in shoot and root length might be due to osmotic stress imposed by salt stress or due to the direct effect of salinity affecting the functioning of photosynthetic apparatus. Several earlier studies reporting significant reduction in vegetative growth and development observed under salt stress screening conditions in Arabidopsis (Karan and Subudhi, 2012b, 2014; Jia et al., 2015), rice (Jain et al., 2006; Shrivastava et al., 2010; Baisakh et al., 2012), maize (Henry et al., 2015), tomato (Žižková et al., 2015), and faba bean (Tavakkoli et al., 2010), were supportive of our findings.

Generally, salt stress during the reproductive stage negatively affect grain yield parameters. The WT plants showed 40 percent seed sterility, which was significantly higher than all transgenic plants under salt stress (**Figure 7A**). Such high seed sterility under abiotic stress conditions could be due to the extreme vulnerability of the pollen development stage during early reproductive and seed maturation stages (Dolferus et al., 2011). Similarly, differences were also observed for grain yield per plant and 100-grain weight. There was a significant increase in grain yield per plant and 100-grain weight only in transgenic pyramided plants than others (**Figures 7B,C**). The lower seed sterility and subsequent improvement in yield parameters observed in transgenic plants under salt stress implied possible mechanism of salt tolerance by preventing excess accumulation of Na^+^ to the developing panicle. The *SaSRP3-1* plants had less seed sterility and more grain yield than *SaVHAc1* plants and likewise *SaVHAc1* plants showed higher 100-grain weight and root length compared to *SaSRP3-1* plants. Interestingly, the results of salt stress revealed a decrease in seed sterility, increased root length, grain yield, and 100-grain weight in pyramided plants compared to the single transgenic plants. The probable explanation for this improved performance of pyramided plants under salt stress condition could be due to the complementary effect of *SaSRP3-1* and *SaVHAc1* genes. Thus, these results support the hypothesis that *SaSRP3-1* and *SaVHAc1* genes function synergistically to impart higher level of salt tolerance. The poor seed setting, low grain weight, and low grain yield observed in control rice plants compared to transgenic plants could be due to alteration of proteins associated with photosynthesis and carbon metabolism under salt stress (Li et al., 2015) and reduced carbon dioxide fixation to stomatal closure (Zhu, 2001; Wang et al., 2015).

In conclusion, our study demonstrated that *SaSRP3-1* and *SaVHAc1* genes function synergistically under salt stress and pyramiding of multiple genes involved in different salt tolerance mechanisms may be a viable approach to achieve higher level of salt tolerance in crop plants. The novel gene *SaSRP3-1* was involved in preventing excess accumulation of Na^+^ and maintaining the structural integrity of plasma membrane under salt stress.

## Author Contributions

PS conceived and supervised the complete study and revised the manuscript. HB and RK conducted the experiments, analyzed the data, and wrote the manuscript. All authors read and approved the manuscript.

## Conflict of Interest Statement

The authors declare that the research was conducted in the absence of any commercial or financial relationships that could be construed as a potential conflict of interest.

## Abbreviations

cDNA: complementary DNA
EST: expressed sequence tag
IPTG: isopropyl β-D-1-thiogalactopyranoside
NCBI: national center for biotechnology information
ORF: open reading frame
PMP: plasma membrane protein
RWC: relative water content
RT-PCR: reverse transcription PCR
TMD: transmembrane domain
WT: wild type
