# Supplementary material for: Transgene Pyramiding of Salt Responsive Protein 3-1 (SaSRP3-1) and SaVHAc1 From Spartina alterniflora L. Enhances Salt Tolerance in Rice
Source: Front Plant Sci. 2018 Sep 12;9:1304. doi: 10.3389/fpls.2018.01304 (PMC6143679; doi:10.3389/fpls.2018.01304)
Supplement: Supplementary file 1 [file Data_Sheet_1.PDF]

Supplementary Materials for

Transgene pyramiding of Salt Responsive Protein 3-1 (*SaSRP3-1)* and *SaVHAc1* from *Spartina alterniflora* L. enhances salt tolerance in rice

Hanamareddy Biradar[1], Ratna Karan[2], Prasanta K. Subudhi[1†],

[1]School of Plant, Environmental, and Soil Sciences, Louisiana State University Agricultural Center, Baton Rouge, LA 70803, USA;[2]University of Florida, Gainesville, FL 32611, USA

[†]Corresponding author. Email: psubudhi@agcenter.lsu.edu

Supplementary Figures S1 to S5

Supplementary Tables S1 to S5

(a)

GGAAGATCTATGCGGGCACGAGTTCTGGATCTGCCTGCTGCTCACTTTCCTCGGCTACATCCCCGGCAT
CATCTACGCCGTCTACGCCATCACCAAATAAGAGATAGCAGCAGCTTCACAAAGCTGTGTTCGTACTCA
AGGATACGTGCAGCTGCAGGTGCCCGGTGGTGATGTGATCGACTTTGTATGAGATCTTGTCTGAGTGCT
CCGTTTCCCGTCCTTCGTGTAATTTT

(b)

M R A R V L D L P A A H F P R L H P R H H L R R L R H H Q I R D S S S F T K L C S Y S R I R A A A G A
R W Stop

(c)

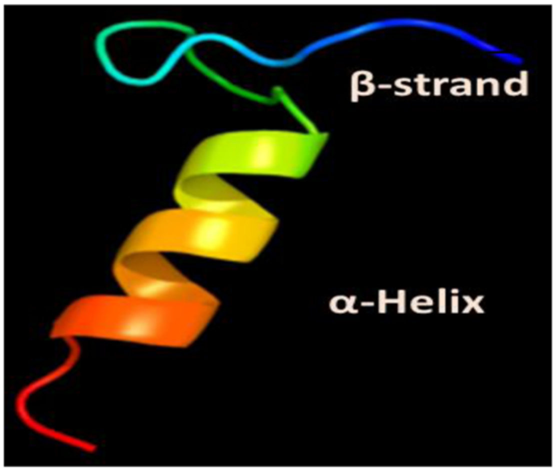

(d)

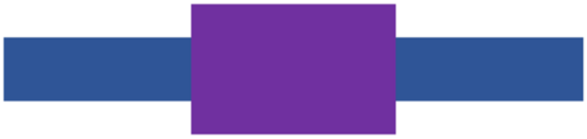

Supplementary Figure S1. Nucleotide and amino acid sequence analysis of *SaSRP3-1*. (a) Nucleotide sequence of *SaSRP3-1* gene of *Spartina alterniflora* L. (Gene Bank Accession number EH277327). The transcriptional start and stop sites are indicated in green and red color, respectively; (b) The deduced amino acid sequences of SaSRP3-1; (c) 3D protein structure model prediction, and (d) Protein domain prediction from the Simple Modular Architecture Research Tool (SMART) program (http://smart.embl-heidelberg.de/help/smart_about.shtml). The purple box indicates the presence of single transmembrane domain from 14th amino acid to 28th amino acid.

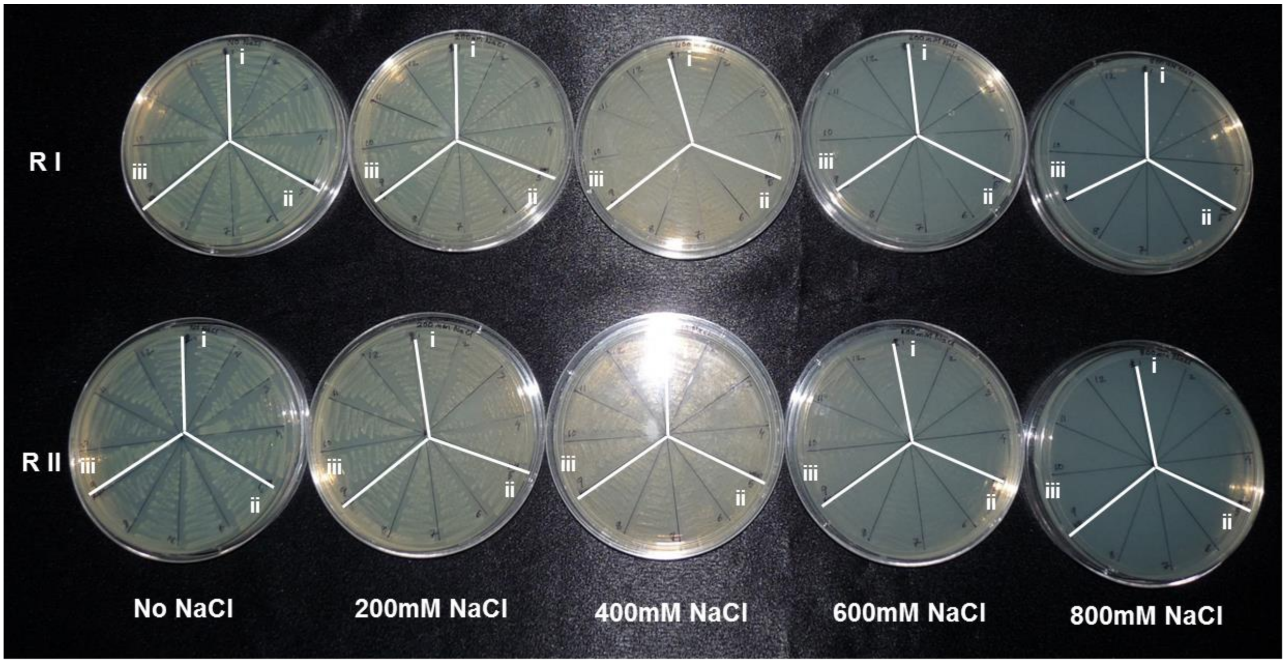

Supplementary Figure S2 Differences in the growth of *E. coli* cells grown under control and different NaCl concentrations. (i) Non transformed *E. coli* (ii) pET-C transformed *E. coli* :Expression control, and (iii) pET-SaSRP3-1 transformed *E. coli*. The experiment was conducted in two replications (R-I and R-II).

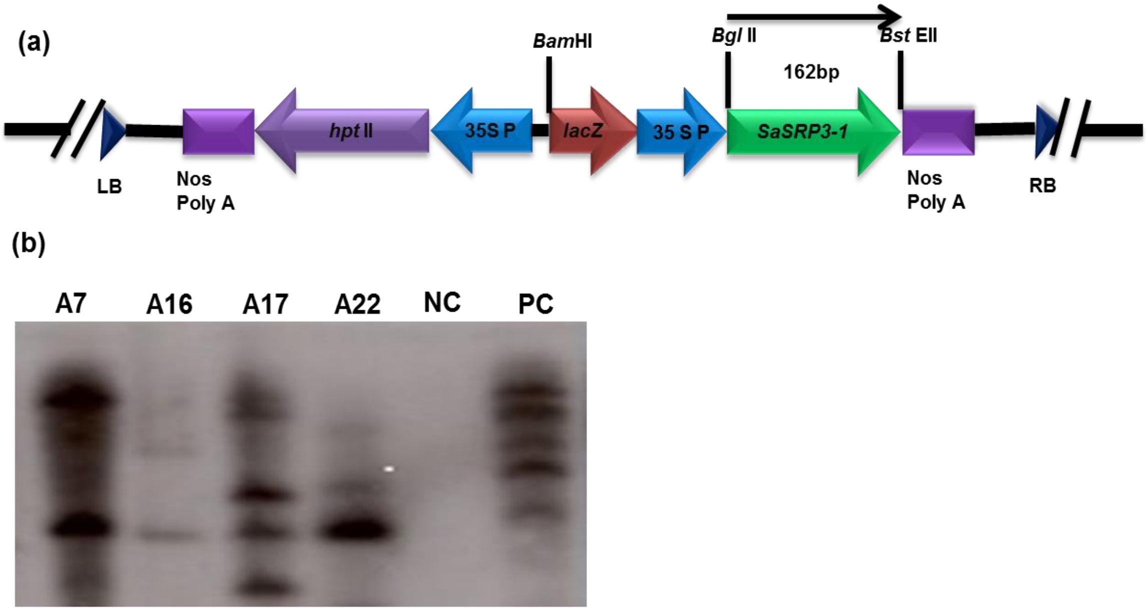

Supplementary Figure S3 Confirmation of the transgene by Southern hybridization. (a) The schematic diagram of the partial linear plasmid vector and position of restriction site *Bam*HI; (b) T_2 *SaSRP3-1* plants confirmation using labeled gene fragment as a probe. A7, A16, A17, A22 : Genomic DNA from individual *SaSRP3-1* T_2 plants digested with *Bam*HI, NC: Negative control (No sample), PC: Positive control of λ phage *Hind*III digested standard.

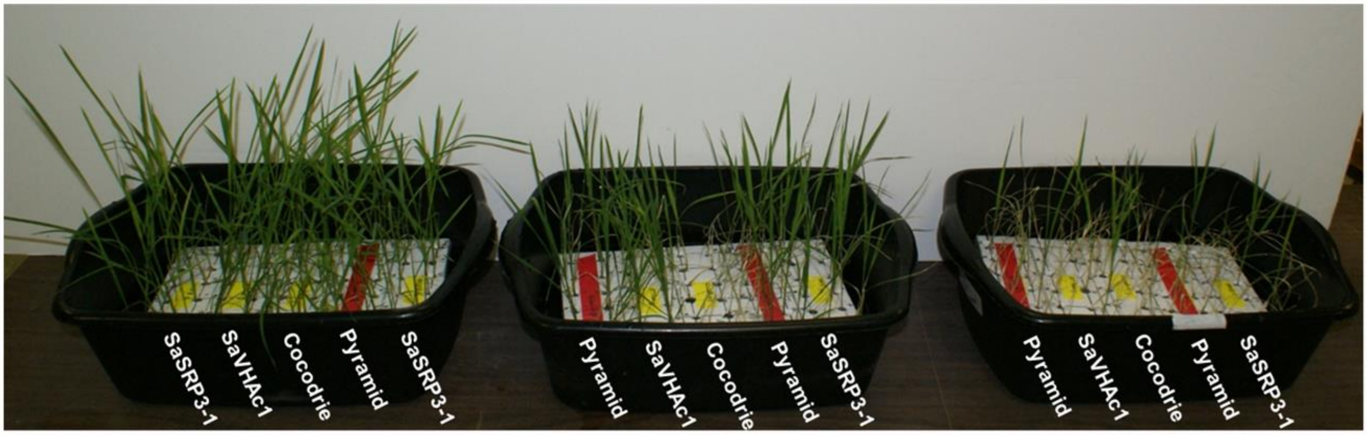

Supplementary Figure S4 Seedling screening for salt tolerance at different levels of salt stress under hydroponic growing condition. Wilting symptoms are visible in Cocodrie (WT) at 100mM NaCl stress.

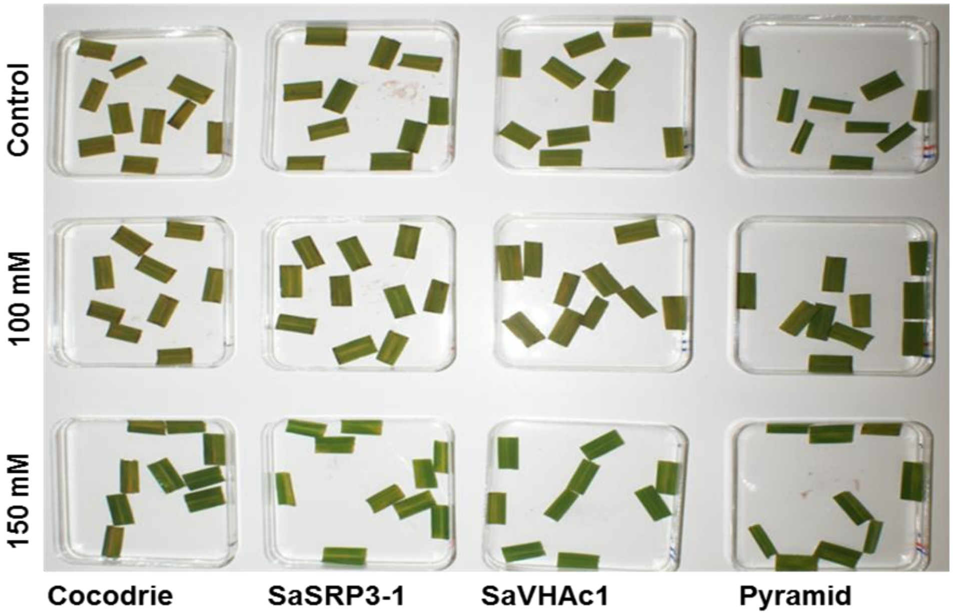

Supplementary Figure S5 Leaf disc assay in Cocodrie (WT), single gene (*SaSRP3-1, SaVHAc1),* and pyramided transgenic rice plants showing salt tolerance 4 days after exposure to different salt concentrations.

Supplementary Table S1 List of primers used in this study

A. Primers for overexpression of *SaSRP3-1* in *Escherichia coli* using pET101/D-TOPO® vector

Primer	5'→3' sequence
SRP3-1pET (F)	5'- CAC CAT GCG GGC ACG AGT TCT GGA T -3'
SRP3-1pET (R)	5'- CCA CCG GGC ACC TGC AGC TGC -3'

B. Primers for cloning *SaSRP3-1* into plant expression vector pCAMBIA 1305.2 and confirming the transgenic plants

Primer	5'→3' sequence
SRP3-1 (F)	5'- GGA AGA TCT ATG CGG GCA CGA G -3'
SRP3-1 (R)	5'- GGG TWA CCT CAC CAC CGG GCA -3'

C. Primers for RT-PCR confirmation of gene transcription

Primer	5'→3' sequence
SRP3-1RT (F)	5'- GAG TTC TGG ATC TGC CTG CT -3'
SRP3-1RT (R)	5'- ACA GCT TTG TGA AGC TGC TG -3'

D. Hygromycin B phospho-transferase (*HPT*) gene specific primers for PCR analysis of transgenic plants

Primer	5'→3' sequence
HPT (F)	5'- TAC TTC TAC ACA GCC ATC -3'
HPT (R)	5'- TAT GTC CTG CGG GTA AAT -3'

E. *SaVHAc1* gene specific primers for PCR analysis of pyramided transgenic plants

PRIMER	5'→3' SEQUENCE
SAVHAC1 (F)	5'- AGG AGG GTG TAC CAT TCG TCA ATG -3'
SAVHAC1 (R)	5'- CCA GGC TCG TAG AGA ATA CCA TTG -3'

Supplementary Table S2 Results from the BLASTN search of *SaSRP3-1* against NCBI nucleotide database.

Accession No.	Description	Maxima score	Total score	Query coverage (%)	E-value	Maxima identity (%)
BT132082.1	*Oryza sativa* clone RRlibD00967 mRNA sequence	141	141	56	1.00E-58	95
AK288109.1	*Oryza sativa* Japonica Group cDNA, clone: J075198O10, full insert sequence	141	141	56	1.00E-58	95
CU406420.1	*Oryza rufipogon* (W1943) cDNA clone: ORW1943C006E13, full insert sequence	141	141	56	1.00E-58	95
CT828904.1	*Oryza sativa* (*indica* cultivar-group) cDNA clone:OSIGCSA031E19, full insert sequence	141	141	56	1.00E-58	95
FJ972825.1	*Cleistogenes songorica* stress-induced hydrophobic peptide mRNA, complete cds	139	139	53	5.00E-71	95
XM_003568926.1	Predicted: *Brachypodium distachyon* hydrophobic protein LTI6B-like (LOC100838178), mRNA	132	132	53	9.00E-28	94
XM_002440512.1	*Sorghum bicolor* hypothetical protein, mRNA	132	132	53	9.00E-46	94
XM_002440511.1	*Sorghum bicolor* hypothetical protein, mRNA	132	132	53	9.00E-46	94
BT062152.1	*Zea mays* full-length cDNA clone ZM_BFb0221E15 mRNA, complete cds	132	132	53	9.00E-46	94
FJ379991.1	*Cupressus sempervirens* isolate cyplp109 putative early drought-induced protein mRNA, partial cds	132	132	53	9.00E-46	94

Supplementary Table S3 Paired t-test results showing the differences in shoot length under control and 100 mM NaCl conditions after 12 days of stress.

Genotypes	Shoot length	DF	t-value	Pr > |t|
Cocodrie	diff	5	7.03	0.0009**
SaSRP3-1	diff	5	5.35	0.0031**
SaVHAc1	diff	5	11.31	<.0001**
Pyramid	diff	5	14.72	<.0001**

*Significance at 5% level; **significance at 1% level; diff: mean difference of genotype before and after stress; DF: degrees of freedom

Supplementary Table S4 Paired t-test results showing differences in root length under control and salt stress (100 mM NaCl) after 12 days.

Genotypes	Root length	DF	t-value	Pr > |t|
Cocodrie	diff	5	2.96	0.0314*
SaSRP3-1	diff	5	0.1	0.9249
SaVHAc1	diff	5	0.28	0.7926
Pyramid	diff	5	-3.08	0.0274*

*: Significance at 5% level, diff: mean difference of genotype before and after stress; DF: degrees of freedom

Supplementary Table S5 Composition of regeneration and rooting media used in this study

Components	Regeneration medium	Rooting medium
MS basal medium	4.4 g/L	4.4 g/L
NAA	0.5 mg/L	0.1 mg/L
Kinetin	0.2 mg/L	No
BAP	2.0 mg/L	No
Sucrose	30.0 g/L	30.0 g/L
Agar	8.0 g/L	8.0 g/L

MS basal medium (*Sigma*, St. Louis) as described by Murashige and Skoog (1962).
